# Highway Improvements

**Published:** 1889-12

**Authors:** 


### HIGHWAY IMPROVEMENTS.

A movement for improving and maintaining better roads throughout the country is beginning to attract considerable attention. While all kinds of business would be increased by good roads, perhaps no class of people can have more interest in this subject than the doctors —unless it should be the doctors' patients. For whatever contributes to assist the doctor in responding with rapidity to the summons of his patients, redounds to their welfare as well as to the health of the community.

The city doctor, especially in Buffalo, has little cause of complaint, but a short ride out of the city, in any direction at the present time, would convince anyone, we think, of the absolute necessity of reform in regard to the building of roads. Very often it is impossible to drive a horse faster than a walk with any hope of getting home with a sound buggy, to say nothing of a whole body. Our country roads, in all but the best summer weather, are so notoriously bad that any attempt to improve them should receive the encouragement of every citizen. The plan seems to be to have uniform laws on the construction and repair and maintenance of all roads within the State, and to this end we understand the attention of the Legislature will be invited during the coming Winter. It is also proposed to bring the matter before Congress with the idea of establishing Inter-State Highways. We trust the influence of all physicians will be exerted on the side of reform, and that they will seek to interest their local representatives in this subject. We have recently received a copy of an address by Col. Albert A. Pope, of Boston, delivered before the Carriage Builders' National Association, at Syracuse, October 17, 1889, calling attention to the importance of highway improvement. Col. Pope also delivered another address on the same subject before the Board of Trade of Syracuse on November 20, 1889. Copies of these addresses can be easily obtained, and they will be found to present much valuable information. If the civilization of a nation is correctly judged by the character of its roads, it behooves this country to bestir itself, for, at present, as Col. Pope says, "The American roads are far below the average; they are certainly among the worst in the civilized world."

